# Visualization of learning-induced synaptic plasticity in output neurons of the *Drosophila* mushroom body γ-lobe

**DOI:** 10.1038/s41598-022-14413-5

**Published:** 2022-06-21

**Authors:** Clare E. Hancock, Vahid Rostami, El Yazid Rachad, Stephan H. Deimel, Martin P. Nawrot, André Fiala

**Affiliations:** 1 Molecular Neurobiology of Behavior, Johann-Friedrich-Blumenbach-Institute of Zoology and Anthropology, University of Göttingen, Julia-Lermontowa-Weg 3, 37077 Göttingen, Germany; 2 Computational Systems Neuroscience, Institute of Zoology, University of Cologne, Zülpicherstraße 47b, 50674 Cologne, Germany

**Keywords:** Neuroscience, Learning and memory, Neural circuits

## Abstract

By learning, through experience, which stimuli coincide with dangers, it is possible to predict outcomes and act pre-emptively to ensure survival. In insects, this process is localized to the mushroom body (MB), the circuitry of which facilitates the coincident detection of sensory stimuli and punishing or rewarding cues and, downstream, the execution of appropriate learned behaviors. Here, we focused our attention on the mushroom body output neurons (MBONs) of the γ-lobes that act as downstream synaptic partners of the MB γ-Kenyon cells (KCs) to ask how the output of the MB γ-lobe is shaped by olfactory associative conditioning, distinguishing this from non-associative stimulus exposure effects, and without the influence of downstream modulation. This was achieved by employing a subcellularly localized calcium sensor to specifically monitor activity at MBON postsynaptic sites. Therein, we identified a robust associative modulation within only one MBON postsynaptic compartment (MBON-γ1pedc > α/β), which displayed a suppressed postsynaptic response to an aversively paired odor. While this MBON did not undergo non-associative modulation, the reverse was true across the remainder of the γ-lobe, where general odor-evoked adaptation was observed, but no conditioned odor-specific modulation. In conclusion, associative synaptic plasticity underlying aversive olfactory learning is localized to one distinct synaptic γKC-to-γMBON connection.

## Introduction

**Figure 1:**
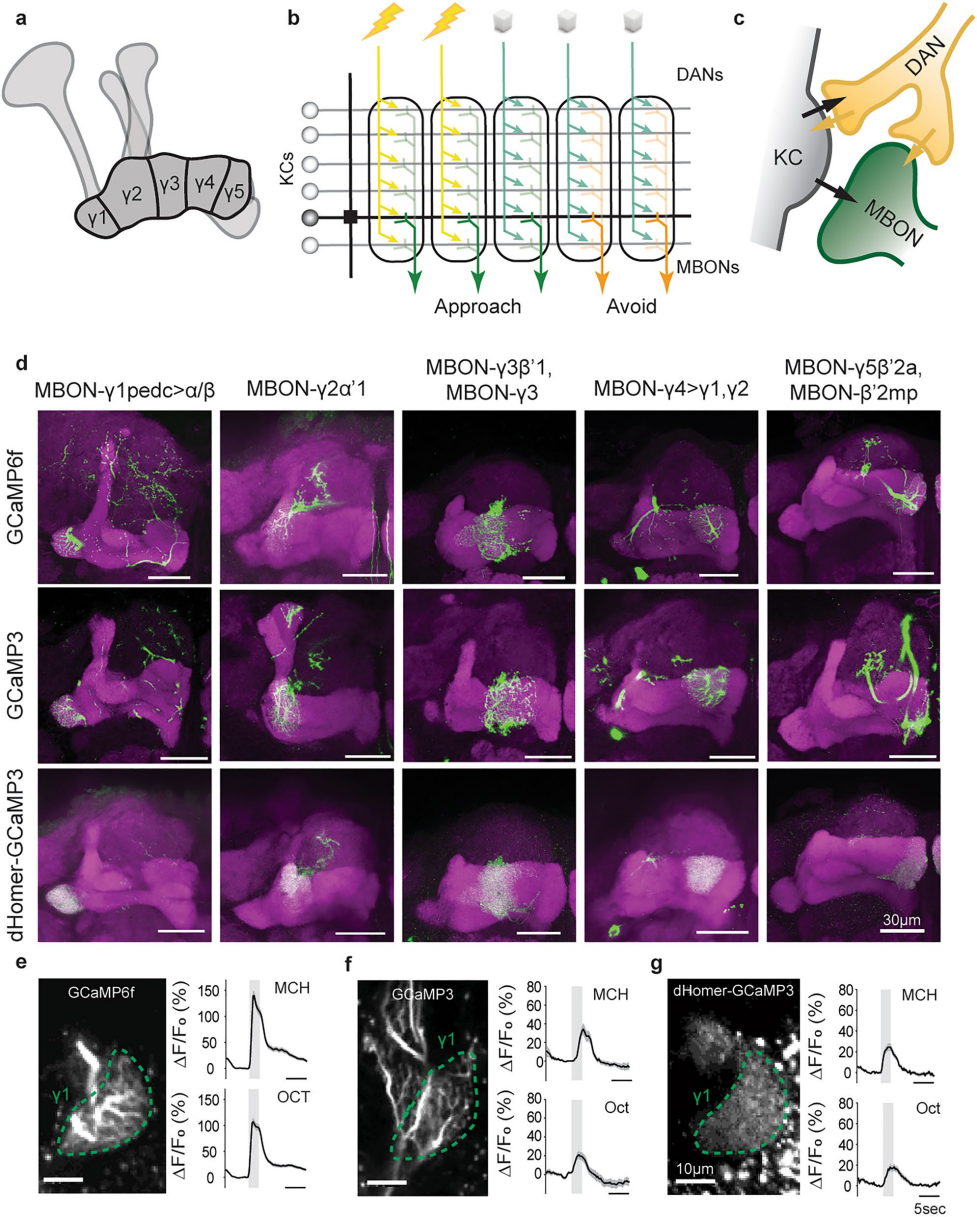
Visualization of postsynaptic calcium in the mushroom body output neurons. (**a**) Mushroom body structure and compartmentalization of the γ-lobe. (**b**) Functional compartmentalization of the γ-lobe, indicating the connectivity between odor-encoding Kenyon cells (KCs), punishminent- or reward-signaling dopaminergic neurons (DANs) and behavior-instructive mushroom body oitput neurons (MBONs). (**c**) Putative connectivity within the Kenyon cell (KC)-dopaminergic neuron (DAN)-mushroom body output neuron (MBON) microcircuit in the γ-lobe. (**d**) Representative confocal images showing the expression pattern of cytosolic sensors GCaMP6f and GCaMP3, and postsynaptically localized homer-GCaMP across the MBONs of the γ-lobe. (**e**–**g**) Examples of functional imaging recordings using cytosolic GCaMP6f and GCaMP3 and postsynaptically localized homer-GCaMP. Line graphs show mean ΔF/F_0_ values and SEM. Grey bars indicate the odor presentation period for the odorants 4-methylcyclohexanol (MCH) and 3-octanol (OCT). For GCaMP6f, n = 119 flies, for GCaMP3, n = 12 flies, for homer-GCaMP, n = 30 flies.

Deciphering how and where in neuronal brain circuits learned information is acquired and stored, i.e., distributed across many neurons or localized to specific synapses, is a central topic in neuroscience. The fruit fly, *Drosophila melanogaster*, has proven to be a valuable model organism for testing hypotheses in this area of research. Indeed, despite their relatively modest brain size compared with mammals, fruit flies are able to perform a variety of complex learning tasks. This includes classical, Pavlovian-style conditioning^1^ in which an odor (conditioned stimulus, CS) is associated with an appetitive or aversive unconditioned stimulus (US)^2,3^. Such associative learning forms the basis of the current study. In *Drosophila*, the mushroom bodies of the central brain have been identified as the site of the neural plasticity that facilitates this learning process^4,5^. More specifically, the γ-lobes of the mushroom bodies are required for the acquisition process and the formation of a short-term memory directly after training^6–8^. Consequently, not only have intrinsic mushroom body neurons (Kenyon cells, KCs) been identified as essential for associative learning, but so too have their extrinsic, axonal up- and downstream synaptic partners. Ensembles of KCs encode different odor stimuli selectively, as sparsely distributed neuronal activity^9–12^. Assigning behavior-instructive values to these odor representations through learning is mediated by axonal upstream US-encoding dopaminergic neurons (DANs) and downstream action-inducing mushroom body output neurons (MBONs)^13–16^. Whereas the former signal the presence of the US at the time of conditioning^15,16^, the latter have been identified as key players in the guiding of approach towards or repulsion away from the learned odor^13^. With distinct populations of MBONs signalling positive or negative valence^13^, current models postulate that the coincident activity of the odor-coding KCs and antagonistically acting (i.e. rewarding or punishing) US-coding DANs leads to a shift in the relative activation of the respective antagonistically acting MBONs^17,18^. This implies that the balanced activity of populations of MBONs ultimately represent the behavior-instructive ‘readout’ of the memory trace. Coupled with findings that learning leads to a synaptically localized modulation of KC activity^11^, and that specifically the synaptic output from the KCs is required for short-term memory recall but not its acquisition^19,20^, the KC-to-MBON presynapses have emerged as the site of plasticity mediating this type of learning^14,17,21,22^. However, determining which exact synapses become modified through the coincidence between the CS and US (i.e., the ‘memory trace’ in a strict sense) has been difficult because electrophysiological or functional imaging approaches typically monitor a neuron’s activity in its entirety, including all excitatory, inhibitory, and modulatory inputs. In this study, we address this issue directly by targeting the genetically encoded calcium indicator GCaMP to the MBON postsynapse, via fusion to the postsynaptic density protein *d*Homer^23^ (Fig. 1). Therefore, the relative changes in fluorescence that we quantify here are reflective of changes in calcium influx directly at the postsynapse, and less so of the integrated calcium signal across the larger dendritic arborizations that lacks the precision required to address our question. Using this more precise tool we were able to ask, for the first time at this level, whether associative plasticity is distributed across different KC-to-MBON synapses along the γ-KC axons or, alternatively, whether it is confined to particular axonal compartments. We found that differential aversive olfactory conditioning led to an odor-specific, associative modulation of a single MBON postsynapse innervating the γ1 compartment. All other MBONs (innervating the γ2-5 compartments) showed non-associative, adaptive plasticity. Therefore, we here confine the associative short-term memory trace to one synapse type along a distinct axonal compartment of the mushroom body. We test this both computationally using a machine learning approach as well as experimentally by identifying the potential downstream consequences of specific, localized plasticity within the MB circuit.

## Methods

### Fly strains

Flies were reared on standard cornmeal food medium at 25 °C and 60% relative humidity with a 12:12 h light/dark cycle. Split-gal4 driver lines used to drive expression in MBONs were obtained from the Bloomington Drosophila Stock Centre: MB112C (BDSC #68263, MBON-γ1pedc > α/β (one neuron per hemisphere)), MB077B (BDSC #68283, MBON-γ2α’2 (two neurons per hemisphere)), MB083C (BDSC #68287, MBON-γ3 and MBON-γ3β’1 (one neuron each per hemisphere)), MB298B (BDSC #68309, MBON-γ4 > γ1γ2 (one per hemisphere)), MB210B (BDSC #68272, MBON-γ5β’2a, MBON-β’2mp (one neuron each per hemisphere)). UAS-GCaMP3^24^ and UAS-GCaMP6f^25^ flies were also obtained from Bloomington Drosophila Stock Centre (BDSC #32236 and #52869, respectively). UAS-dHomerGCaMP3 flies were generated by Pech et al.^23^.

For behavioral experiments using temperature-sensitive UAS-Shibirets to block synaptic output from MBONs, the same split-gal4 driver lines as above were crossed together with UAS-Shibirets or w1118 (both obtained from Bloomington Drosophila Stock Centre, BDSC #5811 and #5905, respectively). The resulting offspring were reared at 18 °C and 60% relative humidity.

### Immunohistochemistry

Flies were cold anaesthetized on ice for 5 min before being moved to a dissection dish and fixed with insect pins. Brains were extracted in Ringer’s solution (5 mM KCl, 130 nM NaCl, 2 mM MgCl_2_, 2 mM CaCl_2_, 5 mM Hepes, 36 mM sucrose, pH 7.3) by removing the head capsule and detaching the brain from the ventral nerve cord. Brains were then fixed in 4% paraformaldehyde at room temperature for 45 min, and then washed three times in PBS with 0.6% Triton-X before being incubated for 2 h at room temperature in blocking solution containing 2% bovine serum albumin. To amplify GCaMP signal, brains were incubated with anti-GFP primary antibody (rabbit anti-GFP, Invitrogen A6455) at a concentration of 1:2000. Additionally, an anti-discs large (DLG) primary antibody (mouse anti-DLG, Developmental Studies Hybridoma Bank 4F3) was used at a concentration of 1:200 to visualize brain structures. Brains were incubated in primary antibody solution for 1 day at 4 °C, and washed again three times in PBS with Triton-X before incubation in secondary antibody. Secondary antibodies anti-rabbit AlexaFluor488 and anti-mouse AlexaFluor633 were used, both at a concentration of 1:300, for 1 day at 4 °C. Brains were washed again three times in PBS with Triton-X, and then mounted in VectaShield for confocal scanning.

### In vivo calcium imaging

Female flies aged between 3 and 8 days after eclosion were prepared and imaged as described by Hancock et al.^26^. Flies were cold anaesthetized on ice for no more than 5 min, placed in a custom-built chamber, and fixed in place using clear adhesive tape. A small window was cut in the tape and the head was glued in position using blue, light-curing glue. The head cuticle was then removed in a drop of Ringer’s solution using a fine-bladed knife and forceps, and excess tissue was carefully removed to expose the brain for imaging. Prepared flies were then placed downstream of a custom-built odor delivery device under a Zeiss 7MP microscope equipped with a Ti–Sapphire laser (Coherent) and a Plan-Apochromat 20× water immersion objective (NA = 1). An excitation wavelength of 920 nm was used. Image acquisition was controlled using Zeiss Zen (2011 SP4) software. Images were captured at a framerate of 4 Hz and with a frame size of 512 × 512 pixels. Simultaneous odor delivery and image acquisition was controlled via a custom written LabView program (National Instruments). Three odors were used in all experiments, as follows: 4-methylcyclohexanol (MCH), 3-Octanol (3-Oct), and 1-Octen-3-ol (1-Oct), at concentrations of 1:750, 1:500, and 1:400, respectively, in mineral oil.

To induce and monitor the effects of aversive olfactory conditioning, a three-stage imaging protocol was used. First, flies were sequentially presented with the three odorants and changes in fluorescence were monitored. Second, the flies were exposed to one of three conditioning protocols. This second step immediately followed the first (i.e., within 60 s). Third, the flies were again presented with the three odorants and their responses were measured (see also the schematic illustration in Fig. 3). This final imaging step was carried out 3–4 min following the end of the respective conditioning protocol. The three conditioning protocols were as follows: classical aversive associative conditioning (‘paired’), in which one odor (the CS+, either MCH or 3-Oct) was presented for 60 s while a pulsing electric shock was delivered to the legs and thorax of the fly (twelve 90 V shocks over 60 s, each lasting 1.25 s), followed by a 60 s break, and then a 60 s presentation of a second odor (the CS−, either MCH or 3-Oct) without the electric shock; an ‘odor only’ control, in which the same procedure was presented with the omission of the electric shock; or a ‘shock only’ control, in which only the electric shock stimulus was delivered, with no odors. In the first and third stages (the ‘pre-training’ and ‘post-training’ imaging steps), all odor presentations had a duration of 2.5 s and were separated by an interval of approximately 40 s. For each MBON type and for each conditioning protocol, 10 individual flies were measured. In rare cases that strong, clear movement artefacts were identified in recordings, flies were removed from analysis.

Images were processed using ImageJ/Fiji (National Institutes of Health, NIH). Any small movements were corrected using the TurboReg plugin^27^. Regions of interest (ROIs) were drawn manually to encompass the entire dendritic compartment for each MBON. The fluorescent intensity throughout the recording period was then extracted from these ROIs and used to calculate the normalized relative change in fluorescence over time (ΔF/F_0_) for each recording. For all measurements, F_0_ was calculated as the average fluorescence intensity over the 2 s preceding odor delivery, and ΔF was calculated by subtracting this value at each time point. In order to statistically compare responses and provide input for the population decoding analysis below, each response was quantified as the area under the curve (AUC) of each ΔF/F_0_ time series. The AUC was derived from the 5 s after odor onset. Due to large variability in odor responses between MCH and 3-Octanol, AUC values were normalized before being used in the population decoding analysis, below (within each experimental group, all responses to each odor were normalized to the mean pre-training response to that odor).

### Population decoding

We devised a supervised machine learning approach with the goal to predict punishment (CS+) vs. no punishment (CS−) from the single trial neural population activity across all five distinct MBON types during the post-training phase of the aversive conditioning protocol (Fig. 5a). This amounts to a binary classification task based on a five-dimensional feature vector for which we devised a support vector machine (SVM).

Because functional imaging could only be performed in a single type of MBON in each individual animal, we had no access to the simultaneous population activity across the five MBON types in the same animal. Per individual and in the post-training phase we are left with two single-trial calcium traces obtained during a single CS+ and a single CS− presentation. For each of the five MBON types we have recordings from nine or ten animals. Thus, we have in total for each MBON type nine or ten per-animal samples (50 samples in total, Fig. 5a) where each consist of one CS+ and one CS− trial. To this end, we used the AUC as normalized time-integrated signal as described above.

For training and testing a single SVM model we proceeded as follows. We randomly drew one sample from each MBON type to define the test set (Fig. 5a). This defines a non-simultaneously recorded pseudo population^28^ of all five MBONs consisting of the population responses to CS+ and CS− (i.e., two vectors that contain five AUC values). The remaining 45 samples (nine per MBON type) represent the training set. For training, we performed 1000 repeated drawings with replacement to obtain a new training sample, i.e. a pseudo population across the five MBON types as illustrated in Fig. 5a. Each sample provided a single feature vector for the class CS+ and a single feature vector for the class CS−. After training a single SVM model with the 2000 feature vectors we tested the model with the two feature vectors of the test set. We denote a correct classification with 1 and an incorrect classification with 0. The possible outcomes for the single model are thus [1, 1], [1, 0], [0, 1] or [0, 0], from which we can compute the single model accuracy as 0%, 50% or 100% with a random chance level of 50%. We repeated the complete procedure for 100 independent models, each defined by the random combination of five samples that constructed the pseudo population of the test set. Across 100 models we then computed the average accuracy in percentage of correct binary classification.

We performed three controls. For each we repeated the same approach as outlined above to obtain mean accuracy and standard error of the mean (Fig. 5b). We first classified single-trial odor responses in the pre-training phase (Fig. 5b). Each single odor response to either MCH or 3-OCT was labeled as CS+ or CS− according to its assigned label during the later training phase. The expected classification accuracy is thus at chance level (50%) before training. We next tested classification during the post-training phase after ‘odor only’ presentation (omission of shock) and during the post-training phase after ‘shock only’ presentation (omission of odor). For both control conditions we created two classes *A* and *B* through random assignment of odors to those labels. We again expect an average accuracy at chance level (50%).

To assess the importance of each individual MBON type for the classification, we repeated the classification approach as explained above by excluding each MBON type one by one from the feature space combination of the four remaining MBON types.

All analysis has been carried out with Python and the implementation of SVM in the Scikit-learn package^29^.

### Classical aversive associative conditioning

To examine the behavioral effects of blocking synaptic output from MBONs during aversive associative conditioning, flies were trained and tested in a custom-built conditioning apparatus. All experiments were carried out at a humidity of 65–85% and either at permissive temperature (22–25 °C) or restrictive temperature (30–32 °C) for control experiments and experiments in which synaptic output was blocked by *Shibire*^*ts*^, respectively.

For the training phase, approximately 30 flies aged between 3 and 7 days were placed into tubes lined with copper wire and connected to an air flow. Flies were conditioned according to the same procedure as the “paired” group described above. Briefly, flies were first presented with an odor (that becomes the CS+ odor) together with a pulsing 90 V electric shock for 60 s. This was followed by a 60 s break before a second odor was presented alone for 60 s (becoming the CS− odor). As in previous experiments, flies were trained reciprocally using the odorants 4-methylcyclohexanol and 3-Octanol. Timely delivery of electric shocks and odor stimuli throughout was controlled by custom-written software.

For the testing phase, flies were transferred from the training tubes to the testing arena. This arena comprised multiple lanes, with each being loaded with one of the groups of approximately 30 flies from the training tubes. Flies were initially held in a central carrier before being released into the lanes, into which the CS+ and CS− odors were presented from opposite sides. The flies were allowed two minutes to distribute between the two sides of the arena before a picture was captured using a camera mounted above the arena that would be used to quantify the distribution of the flies between CS+ and CS−.

To analyze whether flies exhibit a learned avoidance of the punishment-coupled CS+ odor, the number of flies on each side of each lane was counted. Preference indices were calculated independently for reciprocally trained groups (by subtraction of flies that chose CS− from those that chose CS+, divided by total number of flies), before being averaged to generate a learning index. Raw data were processed with OriginPro.

### Statistical analyses

Odor-induced calcium dynamics throughout are presented as line graphs, with lines representing mean ΔF/F_0_ values over time, and shaded areas representing the standard error of the mean (SEM). To quantify and compare responses, the integrated AUC for each ΔF/F_0_ time series was calculated over the 5 s after odor onset. Tests for significant changes in pre-to-post training AUC were carried out using Wilcoxon signed rank tests.

For behavioral experiments (Fig. 6), tests for statistical differences between groups was performed using a one-way ANOVA with a Tukey post-hoc test. A Bonferroni-corrected two-tailed one-sample t-test was also performed on each group to test for differences from 0.

## Results

### Visualization of postsynaptic calcium in mushroom body output neurons

Acquisition and formation of an associative short-term memory is localized to the KC presynapses of the γ-lobe of the MB^6–8^. Thus, when looking for a readout of the synaptic plasticity underlying this form of memory, we focused on the population of compartment-specific MBONs that lie directly downstream of the γ-type KCs. These neurons each receive synaptic input from KCs in their respective compartment, the weight of which is modulated by input from DANs and comprises the canonical site of learning-induced plasticity within this circuit (Fig. 1a–c)^14,17,21^. In addition, MBONs receive direct modulatory input from DANs as well (Fig. 1c). Therefore, we utilized a postsynaptically localized tool, *d*Homer-fused GCaMP3^23^, that allowed for the monitoring of odor-evoked activity precisely at the site of MBON input. To first verify the localization of *d*Homer-GCaMP to the postsynaptic compartments of the MBONs, we removed the brains of flies expressing this construct in individual MBONs of the mushroom body γ-lobe and subjected them to immunohistochemistry and confocal microscopy. When compared to the most often used cytosolic GCaMP6f and the original cytosolic form of GCaMP3, *d*Homer-GCaMP results in a spatially restricted, overall more punctuated fluorescence that is localized primarily in the dendritic compartments of neurons (Fig. 1d). This difference was also observed when neurons were visualized in vivo using two-photon microscopy (Fig. 1e,f), with the large neurites that predominate the GCaMP6f and GCaMP3 fluorescence being absent in *d*Homer-GCaMP-expressing flies (the γ1 compartment innervation by MBON-γ1pedc > α/β is shown here as an example). To further verify that the latter can also be used to detect postsynaptic odor-evoked activity in MBONs, flies were presented with MCH and 3-Oct and changes in fluorescence were quantified (Fig. 1e–g). Indeed, as has been previously documented^25^, odor responses monitored using both cytosolic GCaMP3 and *d*Homer-GCaMP were lower in magnitude observed using GCaMP6f., although both give rise to clear robust odor-evoked changes in fluorescence that can reliably be detected and analyzed. With this, we validate *d*Homer-GCaMP as a viable tool for studying localized odor representations at the level of the MBON postsynapse.

### γ-Lobe MBONs receive heterogeneous odor-evoked inputs

**Figure 2:**
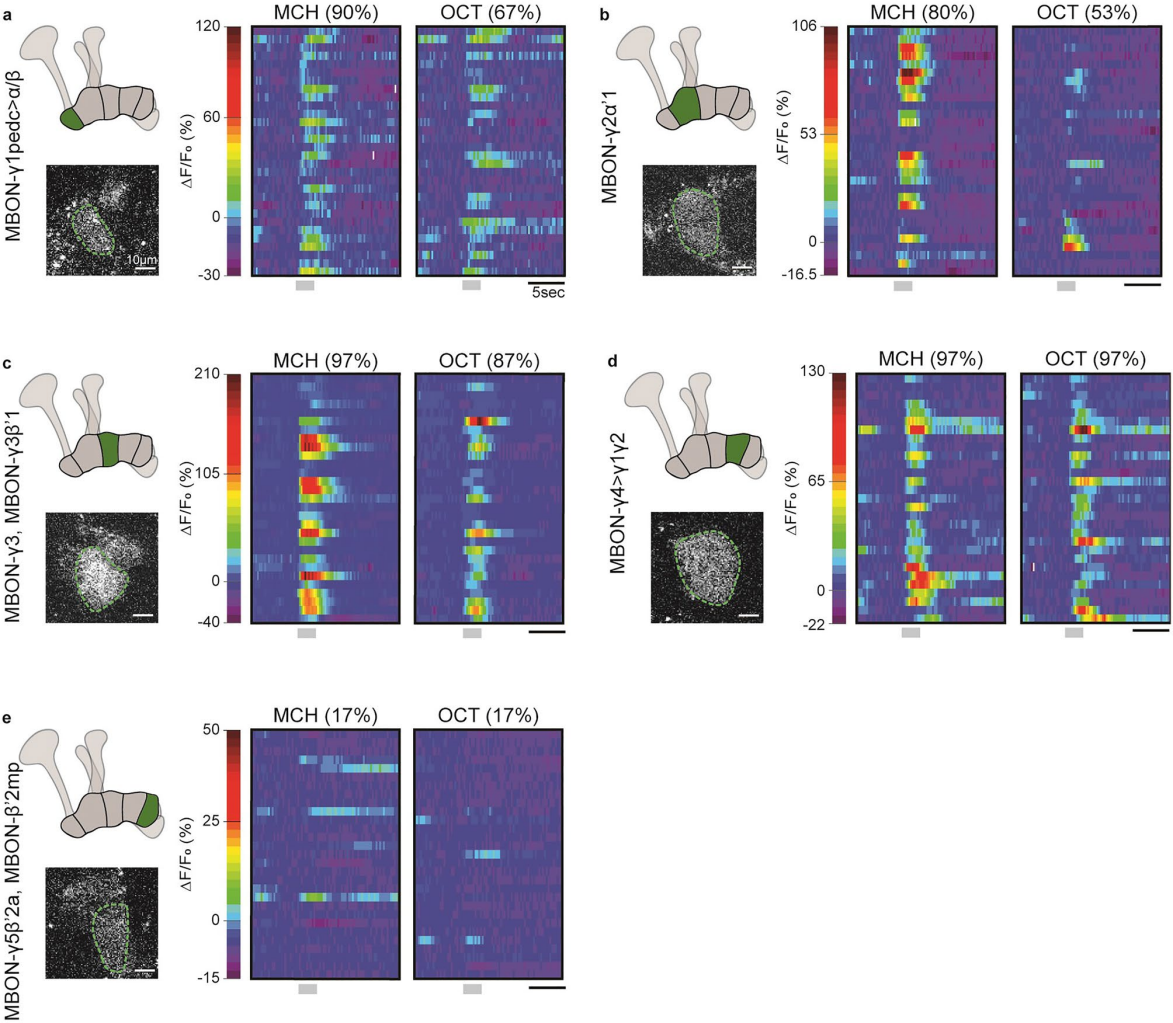
Odor-evoked responses at the MBON postsynapse. Heatmaps show false-color coded ΔF/F_0_ values over time, measured in each of the γ-lobe MBONs ((**a**) MBON-γ1pedc > α/β; (**b**) MBON-γ2α’1; (**c**) MBON-γ3 and MBON-γ3β’1; (**d**) MBON-γ4 > γ1γ2; (**e**) MBON-γ5β’2a and MBON-β’2mp). Each row corresponds to a recording of an individual neuron in an individual fly in response to a single odor presentation. Values above heatmaps show the percentage of flies that showed a response to that odor in each MBON. Grey bars indicate the odor delivery period. For (**a**, **c**–**e**), n = 29; for (**b**), n = 30. Schematic diagrams and single frame in vivo functional imaging examples on the left show the dendritic region of each MBON that was imaged. The green dotted line shows the region that was quantified.

Congruent with their receipt of inputs from large populations of KCs, previous studies have shown that MBONs innervating the MB lobes have broad odor response profiles^30^. In this vein, we first sought to analyze the postsynaptic responsiveness of our γ-MBONs of interest to the experimental odors, MCH and 3-Oct. To do so, we carried out in vivo functional imaging of individual female flies expressing *d*Homer-GCaMP in each of the MBONs and quantified the odor-evoked changes in postsynaptic calcium (Fig. 2; individual and mean response traces shown in Supplementary Fig. 1).

This analysis revealed that, indeed, in the case of the MBONs innervating the γ1, γ3, and γ4 compartments, robust responses to both odors were observable in the majority of flies measured (Fig. 2a,c,d). Surprisingly, the γ2-innervating MBON (γ2α′1) only responded to 3-Oct in a small number of flies, in contrast to MCH (Fig. 2b). It has previously been reported that the dendritic arbors of MBONs in the γ5 compartment (MBON-γ5β′2a, MBON-β′2mp) show little or no response to olfactory stimuli^31^. We also observed this result, with responses being detected in only ~ 17% of flies measured (Fig. 2e, Supplementary Fig. 1). Differences in the magnitude of responses are also notable, with those measured at the MBON-γ3/MBON-γ3β′1 postsynapse sometimes being 4–5 × greater than those measured in, for example, MBON-γ1pedc > α/β. These differences in magnitude were also be observed between odors within animals, with MCH eliciting stronger responses than 3-Oct in most cases. These findings indicate that, first, olfactory responses between the γ-lobe MBONs and, thus, the efficiency of KC-to-MBON connections in different γ-lobe compartments, are not homogenous. Second, within MBON types, responses to different odors are not homogenous across individuals. Therefore, we confirm that the neurons in this MBON population possess distinct and individualized odor response properties that could indeed influence and/or result from individual experience such as olfactory learning, as already reported using a cytosolic calcium sensor^30^.

### Associative conditioning gives rise to compartment-specific plasticity

To investigate if and how these odor representations are influenced by associative learning, we subjected the same flies expressing *d*Homer-GCaMP in the γ-lobe MBONs to an aversive conditioning protocol under the microscope and monitored odor-evoked postsynaptic calcium. Flies were placed in a custom-built imaging chamber in which they could be exposed to both odor and electric shock stimuli during functional imaging, allowing for the visualization of odor-evoked postsynaptic activity before and after an aversive training in which flies learned to associate a given odor with punishment^26^.

Based on the hypothesis that the combinatorial activity of the γ-lobe MBONs holds behavior-instructive information about learned odor valence, we first hypothesized that aversive associative conditioning would lead to bidirectional modulation of MBONs such that approach-mediating MBONs would be suppressed in response to the aversively paired odor and avoidance-mediating MBONs would be potentiated. Indeed, such effects have been reported when measuring with cytosolic calcium indicators or using electrophysiology^22,31–34^. In the following sections, however, we demonstrate that modulation directly at the MBON postsynapse is a highly specialized occurrence, localized to a singular compartment of the γ-lobe.

**Figure 3:**
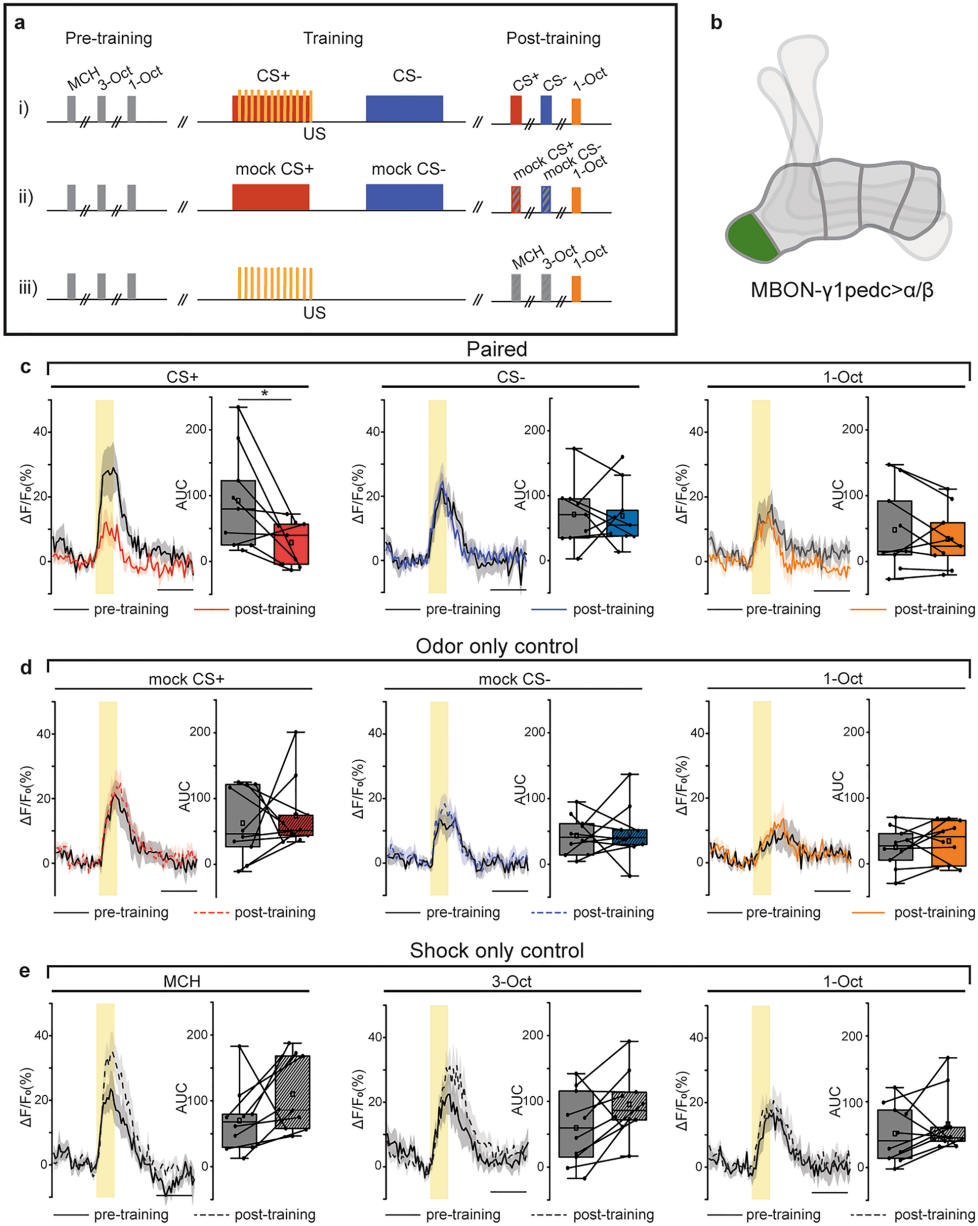
Induction and visualization of learning-induced plasticity at the MBON-γ1pedc > α/β postsynapse. (**a**) Imaging procedures used to probe the effects of associative conditioning on MBON odor responses. Flies were exposed to one of three protocols: (1) aversive associative conditioning; (2) ‘odor only’ control; or (3) ‘shock only’ control. Before and after the training procedure, three odors were presented and MBON postsynaptic calcium was monitored using homer-GCaMP. (**b**) Schematic highlighting the γ1 compartment in which MBON-γ1pedc > α/β postsynaptic sites were imaged. (**c**–**e**) MBON-γ1pedc > α/β odor responses measured before (pre-training) and after (post-training) one of the three training protocols. Lines indicate means, and shaded areas represent SEMs. The black line in each case represents the pre-training response. Post-training responses are shown in color, dependent on condition. The yellow box indicates the period of odor presentation. The area under the curve (AUC) was calculated over the 5 s after odor onset, and pre- to post-training differences were tested using Wilcoxon signed rank tests (*p < 0.05). Boxes represent 25% and 75% quartiles, squares indicate means, and horizontal lines indicate medians. Whiskers show minimum and maximum values. For the paired group (**c**), n = 9 flies. For both control groups (**d**, **e**), n = 10 flies.

Pairing of an odor with electric shock led to a significant reduction in the postsynaptic calcium response elicited by the trained odor (CS+) in MBON-γ1pedc > α/β (Wilcoxon signed rank test, Z = 2.01399, p = 0.03906) (Fig. 3c, left). This is consistent with the identification of this neuron in the signaling of positive stimulus valence^13^. Neither the CS− odor (that was explicitly not paired with electric shock) nor the control odor (1-Oct) elicited statistically significant changes in response after conditioning (CS−: Wilcoxon signed rank test, Z = 0, p = 1; control odor: Wilcoxon signed rank test, Z = 0.82929, p = 0.42578) (Fig. 3c, center and right). This was also the case in the control groups for odor presentation, but without any electric shock (‘CS only’ control; Fig. 3d). This finding demonstrates association-specific modulation of the odor-driven inputs to MBON-γ1pedc > α/β, dependent on CS-US contiguity. Interestingly, the stimulation with electric shock, but without any odor presentation (‘US only control’; Fig. 3e) induced a slight, but not statistically significant increase in MBON-γ1pedc > α/β response at the given sample size. However, when data for both odorants that were also used for associative training, MCH and 3-Oct, were pooled, a statistically significant increase was detected (Supplementary Fig. 3) (Wilcoxon signed rank test, Z = − 2.55729; p = 0.00831). This illustrates that the postsynaptic calcium response in this particular MBON can be bidirectionally modulated; it decreases in response to an odor associated with punishment, and it increases in response to an odor if the punishment does not occur in temporal coincidence with it. Moreover, the enhancement of odor response in this approach-mediating MBON may form part of the physiological basis for the previously described reduction in odor avoidance after electric shock punishment^35^.

**Figure 4:**
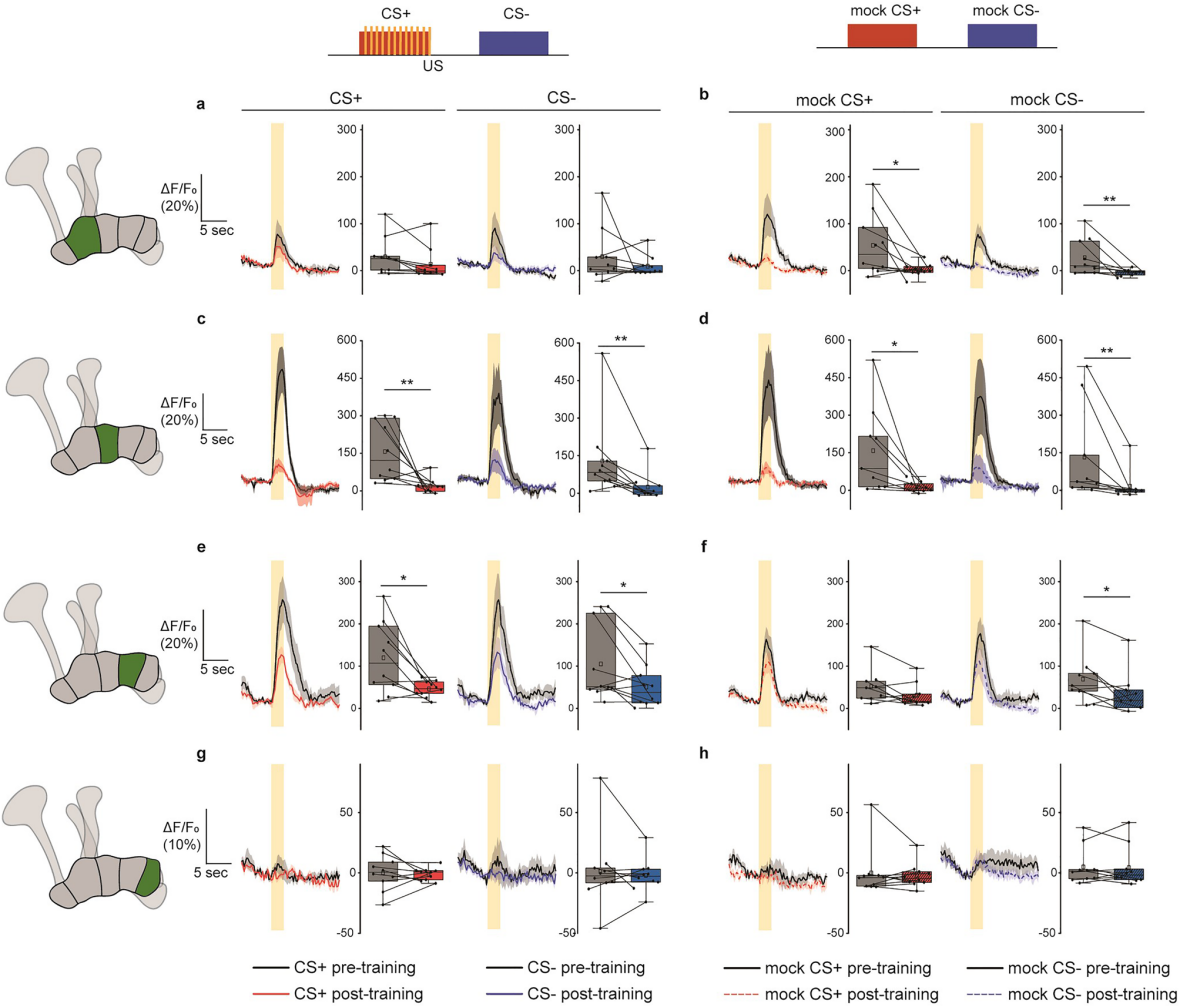
Non-associative plasticity at MBON postsynapses in γ2, 3, and 4 compartments. Odor responses before and after either aversive associative conditioning (**a**, **c**, **e**, **g**) or a control protocol (**b**, **d**, **f**, **h**) in which the unconditioned stimulus was omitted (training protocols, top). In each case, pre-training responses are depicted in black (line graphs) or gray (box plots), and post-training responses are shown in color, dependent on condition. The yellow bar represents the odor presentation period. The area under the curve (AUC) was calculated over the 5 s following odor onset. Boxes represent 25% and 75% quartiles, squares indicate means, and horizontal lines indicate medians. Whiskers show minimum and maximum values. Pre- to post-training effects were tested using Wilcoxon signed rank tests (*p < 0.05; **p < 0.01). For (**a**–**c**, **e**, **g**, **h**), n = 10 flies; for (**d**) and (**f**), n = 9 flies.

Conversely, no association-induced changes were observable in MBONs of the remaining γ-lobe compartments (Fig. 4). Rather, a strong decrease in odor-evoked calcium activity occurred between the pre- and post-training odor response measurements in MBONs innervating the γ3 and γ4 compartments, but both to the CS+ and CS− odor (Fig. 4a,c,e,g). The γ5-innervating MBONs represent an exception in that, in most cases, they showed no responses to odors throughout experiments (Fig. 4g). In MBONs innervating the γ2 compartment, this effect was not statistically significant, perhaps because of relatively weak odor-evoked calcium activity before training in this group of animals (Fig. 4a). The relatively high variability in odor-evoked calcium activity across individuals is in accordance with previous reports that suggest highly individualized, perhaps experience-dependent responsiveness in MBONs innervating the lobes^30^. Flies that received the ‘odor only’ control procedure also displayed strong reductions in responses (Fig. 4b,d,f). Strikingly, the previously high amplitude responses in the γ3-innverating MBON are almost entirely lost through either of these protocols (Fig. 4c,d). We also observed that, although CS+ and CS− -evoked responses underwent similar degrees of adaptation, the γ4-innervating MBON showed statistically weaker depression in response to the “mock CS+” odor in the odor only control condition (Fig. 4e,f). In most cases, this adaptation is not odor-identity specific and is generalized to the third odor, 1-Oct, that is not presented during training (Supplementary Fig. 2). We conclude that this adaptation is likely caused by the prolonged odor exposure that occurs during both the conditioned (paired) and the ‘odor only’ control protocols, as adaptation is much weaker in flies that received the ‘shock only’ control procedure (Supplementary Fig. 3).

These results go beyond confirmation that the MBONs of the γ-lobe show differential naïve odor responses^30^, and that the manner in which those odor responses are modulated by dopamine is diverse across different MBONs^36^. Our data indicate that experience-dependent changes in odor-evoked, postsynaptic calcium activity occur in γ1–γ4 MBONs. However, a differential modulation resulting from CS-US coincidence is restricted to the γ1 compartment. Therefore, the synaptic DAN-KC-MBON microcircuitry that mediates the CS-US association process during aversive olfactory conditioning (i.e., the memory trace in a strict sense) is confined to a single γ-lobe compartment and not distributed across different compartments.

### MBON responses are indicative of whether an odor has been aversively trained or not

**Figure 5:**
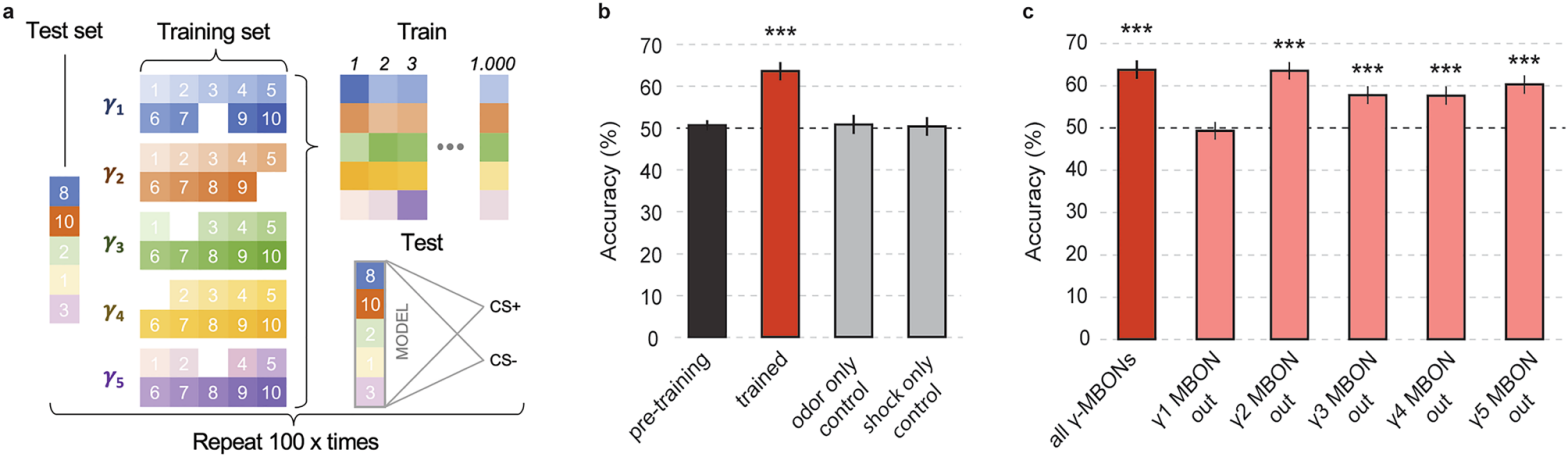
A supervised classifier can distinguish a conditioned from unconditioned odor when γ1-MBON is taken into account. (**a**) Each MBON type (γ1–γ5) was imaged in a group of animals. From this data set we constructed a pseudo-population of non-simultaneously imaged neurons by randomly drawing one individual animal per MBON type. This defined the test set. All remaining sample established the training set. During training, repeated random drawing with replacement allowed to generate independent training samples. Each sample consisted of two single population responses to the presentation of the CS+ odor and to the CS− odor, respectively. After training the SVM model with 1000 such random samples the model was subjected to the previously unseen test set, which again consisted of a single CS+ and a single CS− population response vector. We trained 100 independent models and computed the average accuracy across 100 × 2 classification results. (**b**) Accuracy before and after classical conditioning (‘pretraining’ and ‘trained’) or randomly assigned designations of odor A and B to be differentiated in control conditions (‘odor only’ control and ‘shock only’ control). (**c**) Accuracy after classical conditioning if all γ-lobe MBONs are included, or if one MBON type is removed from the training and test data sets. Bars represent means ± SEM across 100 models. Significant difference from level of chance (50%) determined by one sample Wilcoxon signed rank test (***p < 0.0001).

Given this finding, we sought to test in an unbiased manner whether these observed changes in postsynaptic calcium responses are actually indicative of whether the odor-evoked calcium transients have been aversively trained or not. To do so, we applied a machine learning approach, and used pseudo-populations of γ1–γ5 MBON responses to train a classifier. Over multiple training sessions, the classifier was provided with the pre- or post-training odor responses of such pseudo populations of γ-lobe MBONs and was then asked to predict whether the response was from one of two conditions, CS+ or CS− (Fig. 5a). On average, the conditioned odor (CS+) was distinguished with significantly greater accuracy than any other condition (one sample Wilcoxon sign rank test against level of chance (50%), Z = 16.78943, p = 0) (Fig. 5b). Indeed, all other experimental conditions (pre-training, ‘odor only,’ or ‘shock only’ conditions), resulted in an approximate 50% success rate (one sample Wilcoxon sign rank test against level of chance (50%), pre-training: Z = 1.7353, p = 0.08269, ‘odor only’ control: Z = 0.19191, p = 0.84782, ‘shock only’ control: Z = 1.15577, p = 0.24778) (Fig. 5b), indicating that the odor representations that we observed at the MBON postsynapse are in fact indicators of whether the odor has been trained as aversive or not. We then compared the accuracy with which the classifier could distinguish between CS− and CS+ odors after training in a situation in which one of the five γ-lobe MBON types was removed from the training data sets. Only removing the MBON innervating the γ1 compartment decreased the accuracy of differentiating CS+ from CS− to the level of chance (Fig. 5c) (one sample Wilcoxon signed rank test against level of chance (50%), Z = 1.77029, p = 0.07668). Excluding any other MBON type did not significantly affect the accuracy of discriminability (Fig. 5c) (one sample Wilcoxon signed rank test against level of chance (50%), γ2 excluded: Z = 16.39812, p = 0, γ3 excluded: Z = 9.87016, p = 0, γ4 excluded: Z = 10.72866, p = 0, γ5 excluded: Z = 13.54734, p = 0), in line with our previously drawn conclusion that γ2–γ5 MBON odor responses are not indicative of associative conditioning induced plasticity (despite the slight differentiation in the γ4 MBON between real and “mock” CS+, Fig. 4e,f). This corroborates the finding of the γ1 compartment as the primary site of differential synaptic plasticity underlying aversive discrimination learning.

### Synaptic output from MBON-γ1pedc > α/β and MBON-γ5β’2/MBON-β’2mp instruct behavioral distinction of punished odors

We have provided evidence so far that aversive associative conditioning leads to localized, specific plasticity within a single postsynaptic compartment of the γ-lobe and that this provides a sufficient basis for the distinction of an odor that is predictive of punishment from one that is not. Unclear thus far, however, is if and how this highly localized plasticity relates to behavioral action. Based on recent evidence^13,32,33^ we hypothesize that the CS+-specific reduction in odor-evoked input to the γ1 MBON may lead to a disinhibition of its downstream partners that also instruct behavior, meaning that although the plasticity underlying learned avoidance is restricted to a single compartment, its downstream effects are more broad.

**Figure 6:**
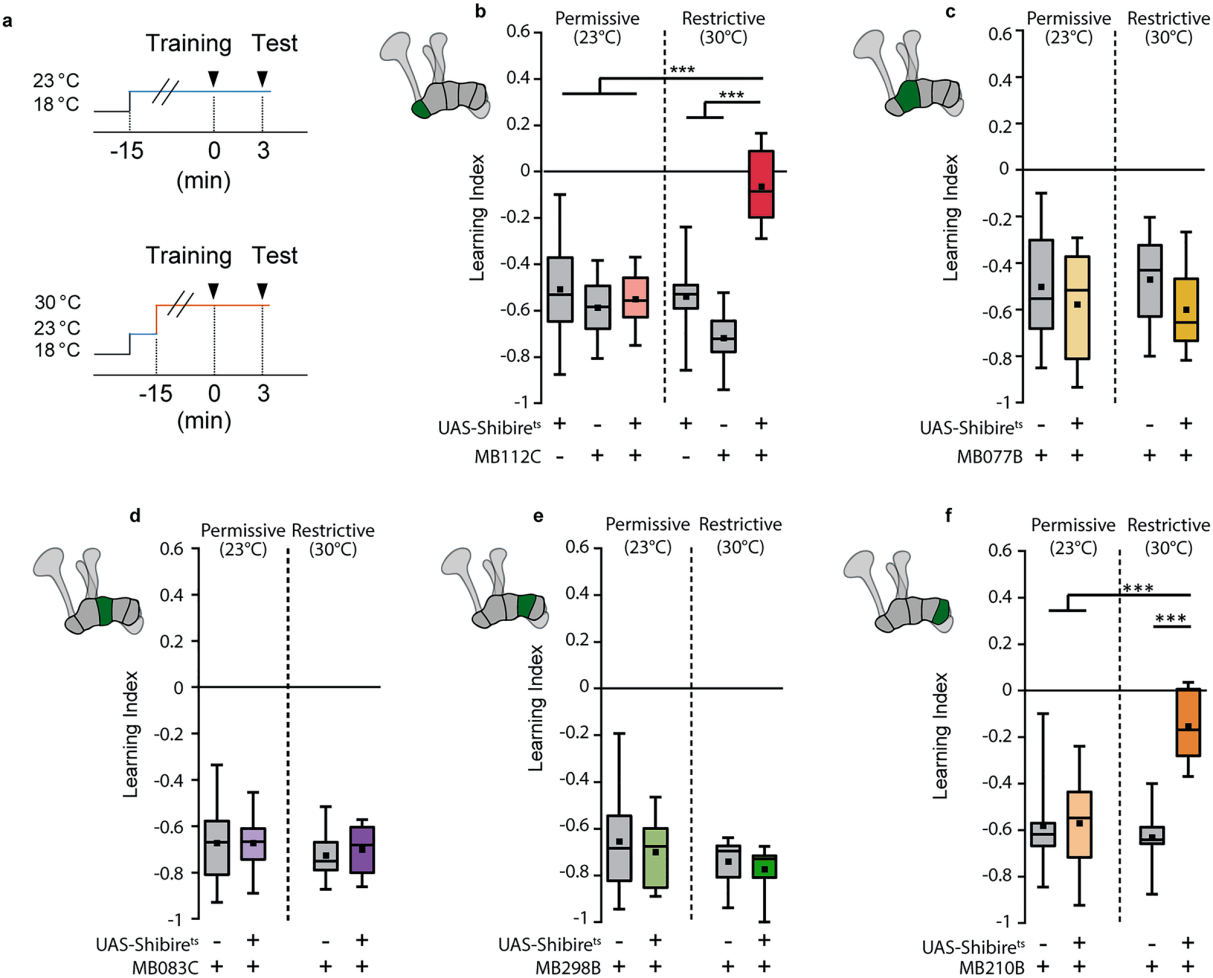
MBONs innervating the γ1 and γ5 mushroom body compartments are required for conditioned odor avoidance. (**a**) Training and test procedures. Training refers to aversive associative conditioning procedure schematized in Fig. 3. Temperature-sensititive shibire expression was used to block synaptic vesicle exocytosis. Upper panel shows the control condition in which training and test were carried out at permissive temperature. Lower panel shows experimental procedure in which training and test were carried out at restrictive temperature. (**b**–**f**) Box plots showing learning indices when blocking synaptic output from different MBON types ((**b**) MBON-γ1pedc > α/β; (**c**) MBON-γ2α′2; (**d**) MBON-γ3, MBON-γ3β′1; (**e**) MBON-γ4 > γ1γ2; (**f**) MBON-γ5β′2a, MBON-β′2mp). Boxes represent 25% and 75% quartiles, squares indicate means, and horizontal lines indicate medians. Whiskers show minimum and maximum values. For all groups, n = 8. Difference between groups were tested using one-way ANOVA with Tukey post-hoc tests.

To assess this theory, we blocked the synaptic output from each of the γ-lobe MBONs using temperature-sensitive shibire^ts^ and tested the flies’ ability to distinguish between an odor that was aversively conditioned (paired with electric shock punishment, CS+ odor) and one that was not (CS− odor)(Fig. 6). Blockage of synaptic output from the γ1 MBON led to a complete absence of conditioned odor preference (Fig. 6b; Bonferroni corrected two-tailed one-sample t-test against test mean of 0, p = 0.3318 [Bonferroni adjusted α = 0.0083). This was not the case for the respective genetic controls or flies trained and tested at permissive temperature. This corroborates previous studies that showed the same effect^13,32^, as well as supporting the indispensability of the γ1 MBON in odor-electric shock conditioning that we concluded from our functional imaging data.

Further mirroring our previous findings, we observed no effect on learning performance when blocking synaptic output from MBONs innervating the γ2, γ3, or γ4 compartments (Fig. 6c–e). The implications of these data are twofold: first, the negligible role of these MBONs in the distinction between odors that are and are not aversively conditioned is consistent between physiological recordings and behavioral observations. Second, associative conditioning-induced modulation of the γ2, γ3, and γ4-innverating MBONs downstream of the KC-to-MBON synapse (e.g., via MBON-to-MBON or DAN-to-MBON connections) is also unlikely. We therefore conclude that, while we do not exclude their importance in other experience-dependent or associative learning tasks, these MBON types play a negligible role in the form of associative conditioning investigated here.

Crucially, we did find that blocking synaptic output from the most distal MBONs of the γ-lobe (innervating γ5 and β′2) caused an inability of flies to distinguish between CS+ and CS− odors when tested for preference (Fig. 6f; two-tailed one-sample t-test against 0, p = 0.031 [Bonferroni adjusted α = 0.0125]). This was not the case for the respective genetic controls or flies trained and tested at permissive temperature. This means that, despite there being no observed plasticity in odor-evoked activity at the postsynaptic sites of these MBONs, they do play a vital role behaviorally. As described above, we suggest that the positioning of these neurons downstream of the γ1 MBON is crucial to this role. The MBONs innervating the γ1 compartment and γ5/β′2 have been shown to convey opposite valences (positive and negative, respectively)^13^, and thus present a logical circuit-level mechanism for the balancing between promotion of approach and promotion of avoidance in response to a conditioned odor. The balance point depends on the strength of KC-to-MBON input in the γ1 compartment and subsequent inhibition exerted by the γ1-innervating MBON on the γ5-innervating MBONs, with both factors determined by the relative (plastic) odor-evoked activity at the γ1 postsynapse.

## Discussion

Determining the substrate mediating the acquisition and storage of learned information through the properties of single neurons, their synapses, and the neural circuits the neurons are part of is a key challenge in neuroscience. Here, we do not define the term ‘memory trace’ as all potential physiological changes within neurons, synapses, and the entire neuronal circuits that accompany or result from the learning process. Considering that plastic changes in synaptic transmission are the principal substrate underlying learning and memory formation^37–39^, we define a memory trace as those synaptic changes that are required and sufficient to induce the learned response^38,40^. In the case of classical conditioning, these synaptic changes must be the site of CS-US integration. Decades of research have established that (a) axonal KC presynapses are required for associative odor learning and short-term memory formation^19,20,40^, and (b) γ-lobe KCs are required in particular^6–8^. The criterion of sufficiency is difficult to address because the stochastic and sparsely distributed nature of odor representations across all KCs precludes any artificial activation of a selected KC and subsequent test of whether an odor stimulus has been learned. However, artificial activation of defined KCs in coincidence with electric shocks has been used to show that a particular KC activity pattern can be trained, and its subsequent activation causes an aversive behavioral response^41^. In addition, plasticity in γ-lobe KCs resulting from associative learning has been clearly demonstrated^11,42^.

However, genetic manipulation of neurons (e.g., using transgenes to depolarize or hyperpolarize neuronal membranes or to block synaptic transmission) inevitably affects entire cells; the question of which dendritic or axonal part of a neuron is required or sufficient for mediating learning remains unanswered using such approaches in isolation. This is of importance because the functional compartmentalization of KC axons, with each compartment being innervated by distinct subsets of DANs mediating reward or punishment, and MBONs inducing different behavioral actions, suggests that entire neurons are not the functional units representing the substrate of memory traces (such as ‘engram cells’). Rather, subcellular partitions of neurons, in this case partitions of axons spanning a compartment, represent independently modulated functional units^11^.

Previous functional imaging and electrophysiological studies recording from neurons in their entirety (i.e., measurements at the soma level), have also reported bidirectional modulation of MBONs as a result of associative olfactory conditioning^22,31,32,34^. We conclude from our data that plasticity in these neurons arises downstream of the KC-to-MBON synapse via previously identified feedforward and feedback loops that exist in the MB circuit. For example, it was shown that plasticity in the specific KC > MBON-γ1pedc > α/β connection does not remain confined to the γ1 compartment, but causes subsequent downstream changes in MBONβ′2mp and MBONγ5β′2a as well via a feedforward inhibition loop^33^. Our behavioral data support the presence and potential significance of such a circuit motif (Fig. 6). As a consequence, flies in which output from either the γ1 or γ5 MBONs is blocked not only during learning, but importantly in the choice situation during retrieval, are unable to correctly prefer the CS− odor over the shock-associated CS+ odor, which confirms previous reports^31^. Note that in Fig. 3 we show that association-driven suppression of γ1 MBON odor responses is specific to only the CS+ and not the CS− odor, while in Fig. 6 MBON output is blocked in the presence of both thus disrupting their distinction in the choice assay. It is of course important to note here that although the vast majority of inputs to MBONs arise from KCs, MBONs receive inputs at various dendritic regions from diverse cell types (including DANs and other MBONs). Our approach to confine the measurement to MBON postsynapses might therefore contribute to distinguishing the primary effects of the CS-US association (i.e., the ‘memory trace’) and subsequent downstream effects that result from it^43^. Indeed, we showcase here the importance of this more precise approach, demonstrating that by looking only at whole cell activity or at crude behavioral output one is at risk of overlooking the true locale and mechanism of the underlying physiology. Importantly, we also used odor stimuli and electric shocks as unconditioned stimuli, as they are typically applied in behavioral learning experiments; this approach is in contrast to optogenetic stimulation of selected DANs^22,36^ that may or may not reflect real sensory stimulation.

Our results directly support recent computational model studies. A circuit model by Springer and Nawrot^18^ assumed downregulation of the synaptic input from KCs to MBON-γ1pedc > α/β through a plasticity mechanism that requires pairing of the CS+ odor stimulus with neuromodulatory input from the aversive US-signaling PPL1-γ1pedc DAN. This simulation showed a significant reduction in MBON-γ1pedc > α/β synaptic input and avoidance behavior after a single pairing, as confirmed in our experiments (Fig. 3). In this model, feedforward inhibition from MBON-γ1pedc > α/β to MBON-γ5β’2a and feedback excitation to dopaminergic neurons establishes a prediction-error mechanism that supports the saturation of the behavioral learning curve and enables extinction learning. Similarly, a recent model by Bennet et al.^44^ assumed the explicit downregulation of the synaptic weight between KCs and an unspecified approach-mediating MBON to implement aversive conditioning and reward prediction through positive MBON-DAN feedback. In fact, recent advances in connectomics have revealed the presence of numerous, multilayered feedback motifs in and around the MB that are implicated in learning and memory formation (see^45–47^). Of course, our data do not rule out the possibility for associative learning-induced modulation of other MBONs in other forms of learning, e.g., over longer time periods, conditioning of different valences such as reward, or context. For example, it is well-established that long-term memory formation requires KCs of the vertical α/α′-lobes^48^. It must be pointed out that our data refer only to the acquisition process and short-term memory formation of aversive olfactory learning.

The broad and generalized suppression of odor responses in all more distal MBON postsynapses indicates that they, unlike MBON-γ1pedc > α/β, possess properties more tuned to signaling non-associative events, such as adaptation resulting from prolonged odor stimulation. This distinction is apparent when comparing postsynaptic responses in flies that received only prolonged odor presentation and those that received that prolonged stimulation together with punishment, with only the γ1-innervating MBON showing a specific depression in response to the punished odor whereas a generalized adaption predominated all other MBON post-training responses. Two key observations indicate that the non-associative changes manifested principally as strong reductions in odor-evoked postsynaptic calcium responses in post-training recordings in the majority of MBONs (namely: MBON-γ2α′1, MBON-γ3 and MBON-γ3β′1, and MBON-γ4 > γ1γ2; see Fig. 4) are likely demonstrative of an intrinsic adaptive property of these MBONs, rather than merely sensory adaptation. First, the observed adapations are not odor specific, but instead are generalized across all odors (Fig. 4, Supplementary Fig. 2). Second, adaptation in the upstream olfactory pathway would theoretically lead to adapation across all MBONs, which we do not observe (MBON-γ1pedc > α/β displayed no adaptation in any context; Fig. 3). This further highlights MBON-γ1pedc > α/β as functionally distinct from the remaining γ-lobe MBONs investigated here. The non-associative effects observed here are yet to be included in future computational circuit models of the fly MB. At the population level of KCs, synaptic plasticity induced by associative olfactory learning causes decorrelation of odor-evoked calcium influx at axonal synaptic boutons, and across the axonal compartments γ2–γ5^11^; axonal KC boutons in the γ1 compartment could not be recorded due to technical limitations, which leaves open the question of whether axonal boutons in this compartment similarly change their correlated activity. However, the fact that differential training affects odor coding across KC populations differentially implies that the learned change in odor-coding of mushroom body circuit and the learned behavior-instructive properties of the MBON signals are two distinct aspects of associative learning that can be experimentally dissociated.

## Supplementary Information

Supplementary Figures.

## Data Availability

The datasets generated during and analyzed during the current study are available in the “Research Data Platform of the DFG Research Unit FOR 2705” repository, http://hdl.handle.net/21.11124/For2705_000095.
