# Supplementary material for: Visualization of learning-induced synaptic plasticity in output neurons of the Drosophila mushroom body γ-lobe
Source: Sci Rep. 2022 Jun 21;12:10421. doi: 10.1038/s41598-022-14413-5 (PMC9213513; doi:10.1038/s41598-022-14413-5)
Supplement: Supplementary file 1 — Supplementary Figures. [file 41598_2022_14413_MOESM1_ESM.pdf]

Supplementary Information

Visualization of learning-induced synaptic plasticity in output neurons of the *Drosophila* mushroom body γ-lobe

Clare E. Hancock[1], Vahid Rostami[2], El Yazid Rachad[1], Stephan H. Deimel[1], Martin P. Nawrot[2], André Fiala[1*]

[1]Molecular Neurobiology of Behavior, Johann-Friedrich-Blumenbach-Institute for Zoology and Anthropology, University of Göttingen, Julia-Lermontowa-Weg 3, 37077, Göttingen, Germany.

[2]Computational Systems Neuroscience, Institute of Zoology, University of Cologne, 50674, Cologne, Germany.

[*]Correspondence: A. Fiala (afiala@biologie.uni-goettingen.de)

Supplementary Figure 1

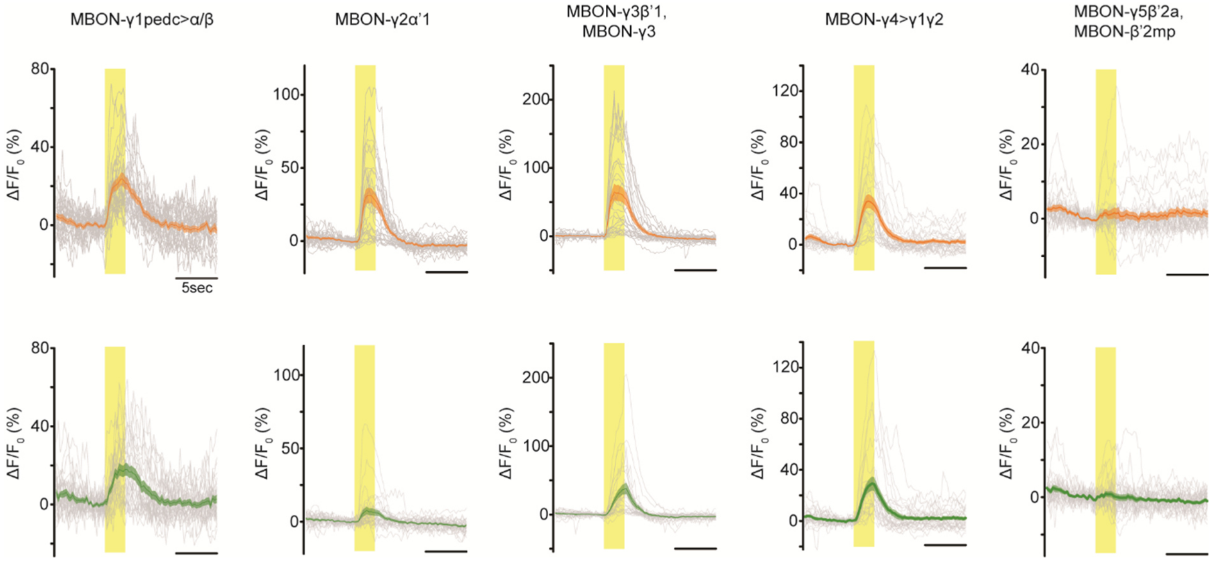

<u>Supplementary Figure 1: Average response traces across γ-lobe MBONs</u>. Average pre-training (naïve) responses to experimental odors, MCH (top row) and 3-Oct (bottom row), for each of the measured MBONs. Colored lines indicate mean responses across individuals, and shaded areas show SEM. Grey lines show single trial response traces for individual flies. The yellow bar indicates the odor presentation period. Sample sizes are as in Figure 2.

Supplementary Figure 2

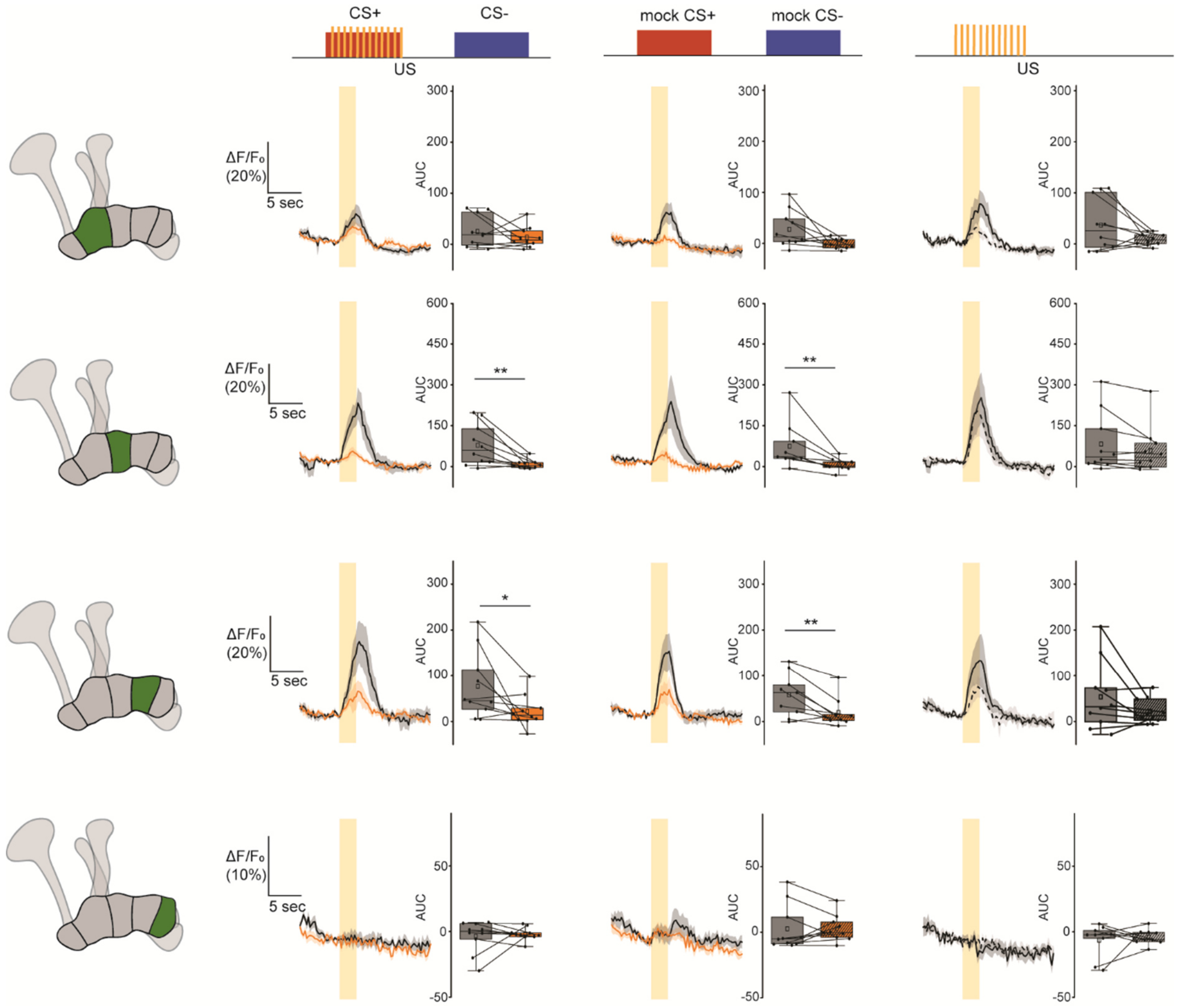

<u>Supplementary Figure 2: Prolonged exposure to experimental odors also leads to generalized adaptation to a third, non-presented odor, 1-Octen-3-ol</u>. MBON responses to 1-Octen-3-ol before and after aversive associative conditioning or control procedures. Boxes represent 25% and 75% quartiles, squares indicate means, and horizontal lines indicates medians. Whiskers show minimum and maximum values. Pre- to post-training effects were tested using Wilcoxon signed rank tests (*p<0.05; **p<0.01). Sample sizes are as in Figure 4.

Supplementary Figure 3

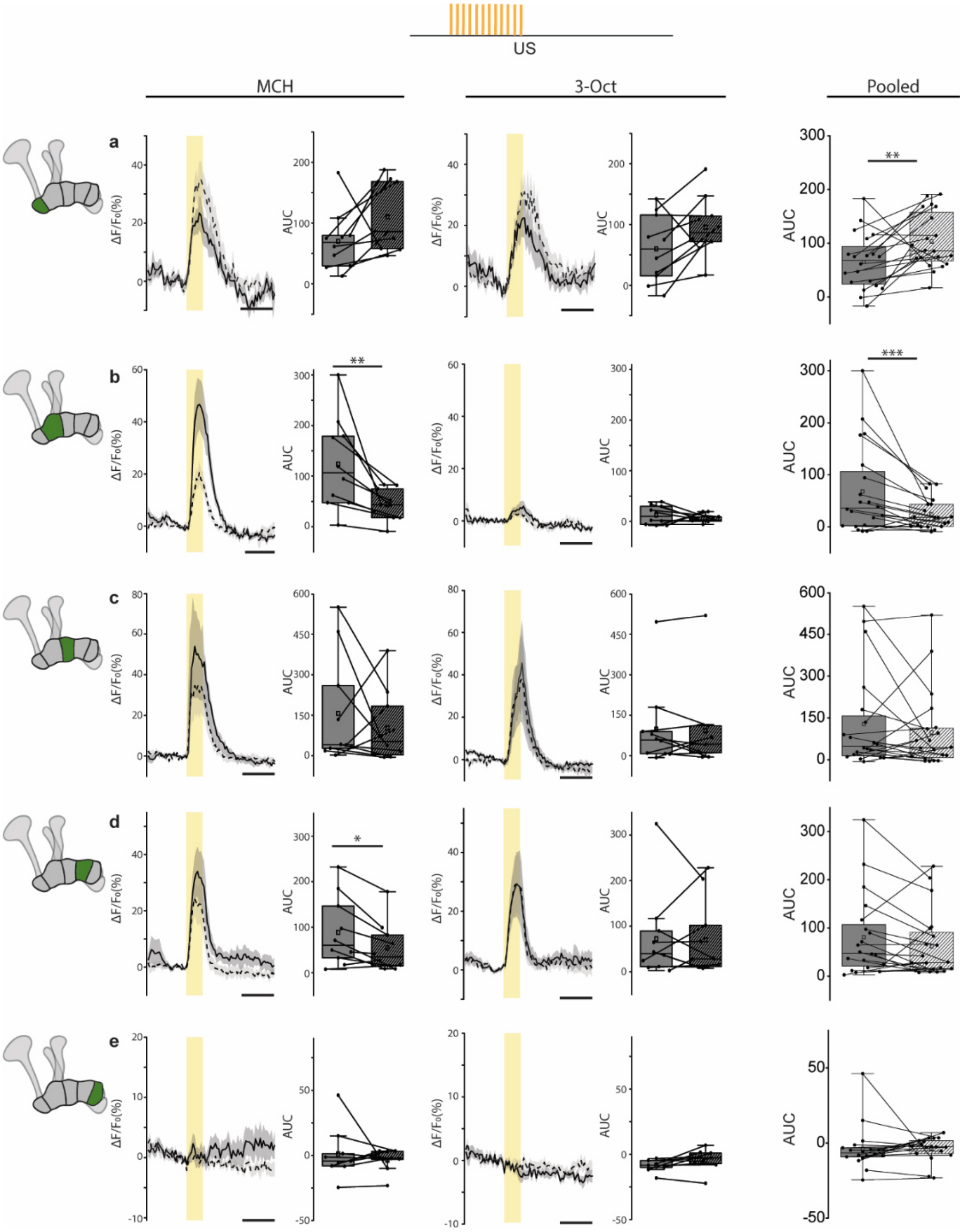

<u>Supplementary Figure 3: Changes in odor-evoked calcium influx in shock only control experiments.</u>

Responses to experimental odors, MCH (left column), 3-Oct (middle column), and pooled together (right column), before and after a 'shock only' control procedure are shown for each MBON. Unpooled data in (a) same as shown in Fig. 3e. Boxes represent 25% and 75% quartiles, squares indicate means, and horizontal lines indicates medians. Whiskers show minimum and maximum values. Pre- to post-training effects were tested using Wilcoxon signed ranks tests (*p<0.05; **p<0.01; ***p<0.001). For a-d, n=20 responses across 10 flies. For e, n=18 responses across 9 flies.
